# Antioxidant Materials in Oral and Maxillofacial Tissue Regeneration: A Narrative Review of the Literature

**DOI:** 10.3390/antiox12030594

**Published:** 2023-02-27

**Authors:** Niloufar Abedi, Zahra Sadat Sajadi-Javan, Monireh Kouhi, Legha Ansari, Abbasali Khademi, Seeram Ramakrishna

**Affiliations:** 1 Dental Materials Research Center, Dental Research Institute, School of Dentistry, Isfahan University of Medical Sciences, Isfahan 81746-73461, Iran; 2 Nosocomial Infection Research Center, Isfahan University of Medical Sciences, Isfahan 81746-73461, Iran; 3 Cellular and Molecular Research Center, Cellular and Molecular Medicine Research Institute, Urmia University of Medical Sciences, Urmia 57157-89400, Iran; 4 Department of Endodontics, Dental Research Center, Dental Research Institute, School of Dentistry, Isfahan University of Medical Sciences, Isfahan 81746-73461, Iran; 5 Department of Mechanical Engineering, National University of Singapore, 9 Engineering Drive 1, Singapore 117576, Singapore

**Keywords:** cranio-maxillofacial, dental, reactive oxygen species, antioxidants, tissue regeneration

## Abstract

Oral and maxillofacial tissue defects caused by trauma, tumor reactions, congenital anomalies, ischemic diseases, infectious diseases, surgical resection, and odontogenic cysts present a formidable challenge for reconstruction. Tissue regeneration using functional biomaterials and cell therapy strategies has raised great concerns in the treatment of damaged tissue during the past few decades. However, during biomaterials implantation and cell transplantation, the production of excessive reactive oxygen species (ROS) may hinder tissue repair as it commonly causes severe tissue injuries leading to the cell damage. These products exist in form of oxidant molecules such as hydrogen peroxide, superoxide ions, hydroxyl radicals, and nitrogen oxide. These days, many scientists have focused on the application of ROS-scavenging components in the body during the tissue regeneration process. One of these scavenging components is antioxidants, which are beneficial materials for the treatment of damaged tissues and keeping tissues safe against free radicals. Antioxidants are divided into natural and synthetic sources. In the current review article, different antioxidant sources and their mechanism of action are discussed. The applications of antioxidants in the regeneration of oral and maxillofacial tissues, including hard tissues of cranial, alveolar bone, dental tissue, oral soft tissue (dental pulp, periodontal soft tissue), facial nerve, and cartilage tissues, are also highlighted in the following parts.

## 1. Introduction

Oral and craniomaxillofacial diseases in the area of the face, dental arches, and craniofacial hard and soft tissues are caused by chemical, physical, microbial factors, and systemic diseases [1]. Among these diseases are oral infectious diseases such as dental caries and periodontitis; craniofacial tissue disorders (due to cysts, tumors, trauma, and abnormalities); and other conditions, including salivary gland disorders, neurological diseases, and temporomandibular joint (TMJ) disorders, which are usually related to teeth [2]. Some clinical strategies are available for treating damaged tissue, such as autografting, allografting, and xenografting; however, none of these methods is the ideal solution due to their drawbacks and limitations [3,4]. Multifunctional biomaterials have been proven to be a promising alternative treatment strategy [5]. However, in practice, implanted biomaterials face some problems, such as reactive oxygen species (ROS) and free radicals generated by immune responses and surrounding connective tissue, such as hydrogen peroxide (H_2_O_2_), superoxide ions (O_2_^−^), hydroxyl radical (OH·), and nitrogen oxide (NO), which can interfere with successful cellular repopulation and tissue regeneration following transplantation [6,7]. While oxidative stress at low concentrations has many physiological functions and plays an important role in tissue regeneration [8], the high level of ROS may hinder tissue repair as it commonly causes severe tissue injuries, leading to cell damage through distinct mechanisms, such as membrane disorganization and protein/nucleic acid damage [9,10]. Tissue-engineered implants are susceptible to increasing ROS levels because of the destruction caused by reperfusion after ischemia, which occurs in tissue and organ engineering transplants [11,12].

Additionally, tissue oxygenation during in vitro implant preservation and surgery may lead to local hyperoxia and contribute to ROS-induced organ damage [13,14]. Consequently, it has been proposed that delivering antioxidant materials combined with tissue engineering may be viable to achieve efficient tissue/organ regeneration and limit post-implantation failure. Specific kinds of antioxidant materials in the form of micro/nanosized particles (e.g., ceria (nanoceria)), carbon materials (e.g., carbon nanotubes), manganese, and selenium [15,16,17] and medicinal herb extracts (e.g., enzymatic (catalase and glutathione peroxidase)) and non-enzymatic antioxidants (flavonoids and polyphenols) have been successfully applied to scavenge free radicals and prevent excess ROS generation in tissue engineering and regenerative medicine applications [18,19,20]. There is an emerging trend of using antioxidants as bioactive, ROS-reducing molecules to modify implants/tissue-engineered grafts. The ability of these biomolecules to control oxidation-reduction (redox) processes in vivo and in vitro (3D cell culture before surgical implantation) can facilitate cell survival and homeostasis. Overall, understanding the fundamental concepts of antioxidants and their integration with tissue regeneration principles is crucial for the successful inclusion of antioxidant materials into regenerating tissue in the oral and maxillofacial areas. This review mainly emphasizes the intersection of antioxidant materials and oral and maxillofacial tissue regeneration. To this aim, a brief description of antioxidant materials, their classification, and their mechanism of action is provided. Then, the review discusses the applications of antioxidants in the regeneration of oral and maxillofacial tissue, including hard tissues of cranial, alveolar, dental tissue, oral soft tissue (dental pulp, periodontal soft tissue), and facial nerve and cartilage tissues by searching the five popular databases: PubMed, Scopus, Web of Sciences, Cochrane, and Google Scholar up to December 2022, using relevant keywords. Inclusion criteria were studies that were written in English, and discussed the effect of at least one antioxidant material in any form on oral and maxillofacial tissue regeneration. 

## 2. Antioxidant Materials

Antioxidants are materials that preserve cells from the damages caused by free radicals [21]. They can control the harm of oxidative stress directly through the reaction with free radicals or indirectly through inhibition of the activity of free radical-producing enzymes or improvement of the activity of intracellular antioxidant enzymes. In addition to the inhibitory impact of antioxidant materials on ROS products, their osteogenic/odontogenic differentiation effects are of great interest in dental and facial tissue regeneration. For instance, the literature has reported that the cocktail of dexamethasone (Dex), ascorbic acid (Asc), and β-glycerophosphate (β-Gly) could enhance the osteogenic differentiation of stem cells by intracellular signaling cascades regulation [22]. It was shown that Asc could increase the secretion of collagen type I (Col1), thereby affecting the Col1/α_2_β_1_ integrin-mediated signaling. Similarly, β-Gly was reported to act as a source of phosphate in hydroxylapatite formation in bone structure [22]. Moreover, some exogenous antioxidants affect the odontoblast-like cells to differentiate from dental pulp stem cells by expressing the odontoblast markers dentinal sialophosphoprotein and dentin matrix protein-1 concomitantly with RUNX2 transcripts [23,24,25]. For example, quercetin is a suitable source for the odontogenic differentiation of dental pulp stem cells by increasing alkaline phosphatase (ALP) activity and the expression of dentin sialophosphoprotein [26,27]. Other classes of naturally occurring compounds, such as anthocyanins, have great osteogenic effects in healing bone disorders by upregulating osteoblastic genes, promoting the proliferation of osteoblasts, enhancing mineral nodule formation, and protecting against bone mass loss in osteopenic conditions [28]. Retinoic acid and ascorbic acid, as natural sources of antioxidants, have also shown a significant osteoblastic effect on human dental pulp mesenchymal stem cells. They can increase RUNX2 gene expression and form the calcified nodules in cells [29].

**Figure 1:**
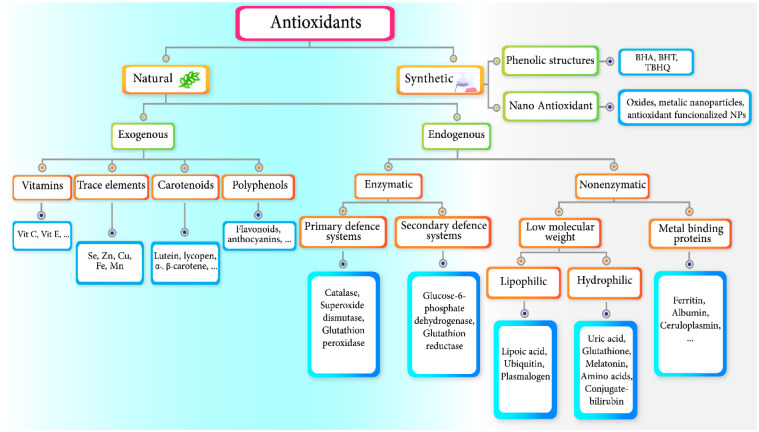
Summary of antioxidant classification.

There are two main classes of antioxidants, depending on their source: natural and synthetic. The diagram of the antioxidant classification is presented in Figure 1.

### 2.1. Natural Antioxidants

Plants, such as edible fruits, vegetables, spices, and herbs, are the main source of natural antioxidants because they are rich in polyphenols, carotenoids, vitamins, and minerals [30,31]. Natural antioxidants can hinder the approaches of oxidation and the microorganism’s growth [32]. As depicted in Figure 1, there are two major categories of natural antioxidants, i.e., exogenous and endogenous antioxidants. While endogenous antioxidants are responsible for repairing all of the damages caused by free radicals via initiating cell regeneration from the inside on out, exogenous antioxidants only repair some of the damages caused by free radicals outside and inside by stimulating (not initiating) cell regeneration. The endogenous antioxidants are divided into enzymatic and non-enzymatic antioxidants. They are different in place and mode of action and final effects. Enzymatic antioxidants mainly protect the cells, while non-enzymatic antioxidants generally act in the plasma. Enzymatic antioxidants are uniquely generated by the human body and as indicated in Figure 1 and are classified into primary and secondary antioxidants. Primary antioxidants are as follows: catalase (CAT), superoxide dismutase (SOD), and glutathione peroxidase (GPx) [33]. Because of its vital role in maintaining cellular redox homeostasis, glutathione (GSH) is one of the essential cellular antioxidants. The cysteine amino acid contains a thiol group, which is a reducing agent, and regularly and repeatedly undergoes a reversible oxidation-reduction reaction. The cells preserve a high concentration of the reduced form of glutathione with the help of glutathione reductase (GR). GSH, in turn, can reduce other enzymes and metabolites [33]. Nonenzymatic antioxidants are not found in the human body naturally and must be supplemented in a diet for proper metabolism [34]. Some of the known nonenzymatic antioxidants are trace elements, vitamins, polyphenols, carotenoids, and other antioxidants. In addition to these nonenzymatic exogenous antioxidants, there are some nonenzymatic endogenous antioxidants such as albumin, lipoic acid, uric acid, glutathione, amino acids, and etc. For example, albumin is a certain protein that can bind to endogenous compounds and metal ions. Accordingly, this versatile protein is involved in many biochemical processes, such as antioxidant defense, as it can decrease the extracellular component of oxidative stress. The important point to note is that the antioxidant property of albumin can be affected by its reactions with active species [35]. α-Lipoic acid and dihydrolipoic acid reveal direct free radical scavenging properties, as it is evidenced that α-lipoic acid supplementation reduces oxidative stress and reforms decreased levels of other antioxidants [36]. Similarly, it was revealed that uric acid contributes to antioxidant reactions in the human lung by scavenging HO and HOCI [37].

### 2.2. Synthetic Antioxidants

Synthetic antioxidants are molecules of various chemical structures created by specialists to benefit humanity. These antioxidants have been successfully used in several products, including food, pharmaceuticals, and cosmetics; however, their carcinogenic activity has limited their usage [38]. Butylated hydroxy anisole (BHA), butylated hydroxytoluene (BHT), propyl gallate (PG), tert-butyl hydroquinone (TBHQ), and ethylenediaminetetra acetic acid (EDTA) are the most commonly utilized synthetic antioxidants. Synthetic antioxidants are bioequivalent to their natural counterparts; for example, biovitamin C vs. chemically manufactured L-ascorbic acid [21,39]. These antioxidants are often more active and purer than natural antioxidants and have consistent antioxidant activity; however, they must meet the regulatory authorities’ nontoxicity and safety criteria before marketing. By using natural-identical antioxidants, we can combine the benefits of both entirely synthetic (low cost, high activity, stability, and reproducibility) and natural (healthy) antioxidants. 

## 3. Mechanisms of Antioxidants’ Activity

The mechanisms of the antioxidant compounds’ activities have a close relationship with the chemical structure of the free radicals, the reactivity of the free radicals, and the environment where the reactive species are found. Hence, describing the ROS and reactive nitrogen species (RNS), which include both free radicals and precursors, is very important. It should be mentioned that the reaction between the free radicals and antioxidants is a second-order reaction; therefore, it not only depends on the amount of free radicals and antioxidants but also depends on the chemical structure of both reagents, reaction conditions, and medium. For instance, phenolic structures inhibit or reduce free radicals through hydrogen atoms transferred from their hydroxyl group. The mechanism of the reaction of a peroxyl radical (ROO^•^) containing phenolic antioxidants involves a concerted transfer of the hydrogen cation from the phenol to the radical, forming a transition state of an H-O bond with one electron. The capacity of phenolic antioxidants is highly reduced when a solvent prone exists in the reaction medium to form a hydrogen bond with the phenolic antioxidants. Another antioxidant component, vitamin C, chemically reacts with the important ROS in the body and acts as a hydrosoluble antioxidant. The mechanism of its antioxidant activity is associated with hydrogen atom transfer to peroxyl radicals, molecular oxygen elimination, and inactivation of singlet oxygen [40,41]. Many chemical and/or biological research models have been established to evaluate the concept of mechanism of the antioxidant activity. The main free radicals commonly used to measure the radical scavenging ability of antioxidants along with their main mechanism are discussed here.

### 3.1. Free Radical Scavenging

The radical scavenging mechanism is dependent on the use of either O_2_^−•^ and ^•^OH, or 1,1-diphenyl-2-picryl-hydrazyl (DPPH), 2,2-azino-bis (3-ethylbenzthiazoline-6-sulfonic acid) (ABTS˙+), galvinoxyl, and N, N-dimethyl-p-phenylenediamine dihydrochloride (DMPD˙+). DPPH and ABTS˙+ scavenging methods have been the most frequently used to assess the antioxidant activity of materials due to their easy, fast, sensitive, and reproducible procedures. 

#### 3.1.1. Scavenging Superoxide

O_2_^−•^ is a main cellular free radical, and its scavenging is a well-known model for identifying the activity level of antioxidant compounds [42]. Mainly, O_2_^−•^ can be produced during the mitochondrial respiration process as a byproduct. In addition, in phagocytes, O_2_^−•^ is generated in considerable amounts by the NADPH oxidase enzyme for the cleanup of pathogens. While O_2_^−•^ itself is not so dangerous to biomolecules, it contributes to producing more reactive OH˙ and ONOO^−^ [43]. Many probes and techniques, including spectrophotometric (nitro blue tetrazolium (NBT)), chemiluminescent (lucigenin, coelenterazine, …), enzymatic (cytochrome c, aconitase), fluorescent (dihydroethidium, fluorescein, rhodamine, and MitoSOX), and electron paramagnetic resonance spin trapping have been developed for the detection of O_2_^−•^ production [44].

#### 3.1.2. Scavenging Hydroxyl Radical

Hydroxyl radical (^•^OH) is highly powerful and more dangerous than other free radical species which can damage biological components, including DNA, lipids, and proteins. According to most beliefs, ^•^OH is produced in the Fe^2+^ (or Cu^+^)/H_2_O_2_ Fenton reaction system via the incubation of FeSO_4_ and H_2_O_2_ in an aqueous solution. Thus, direct scavenging or the prevention of ^•^OH generation through free metal ion chelation or H_2_O_2_ conversion to other harmless structures is the way to the accomplishment of the ^•^OH scavenging activity of antioxidants.

A type of spectrophotometric probe for determining ^•^OH is the Fe^3+^-EDTA-H_2_O_2_-deoxyribose system. ^•^OH, generated in this system degrades deoxyribose to a reactive species, malondialdehyde, which subsequently forms a compound with thiobarbituric acid (TBA). The changes in the absorbance wavelength of MDA–TBA at 532 nm is an indicator for the assessment of the antioxidant ability for ^•^OH scavenging [45,46]. In another method, using electron paramagnetic resonance (EPR) spin trapping, 5,5-dimethyl-1pyrroline-N-oxide (DMPO) reacts with ^•^OH and forms the DMPO-OH radical, which can be monitored by EPR. The changes in EPR intensities of the DMPO-OH radicals are an indicator for the assessment of antioxidant ability for radical scavenging [47].

#### 3.1.3. More Stable Radical Scavenging

Radicals, including galvinoxyl, ABTS˙+, DMPD˙+, and DPPH, are colored and more stable radicals compared to naturally occurring radicals, which sparked intensive research interest. While DPPH and gavinoxyl radicals are commercially available, ABTS˙+ and DMPD˙+ radicals can be prepared immediately before their use [48]. The strong absorption peak of these radicals in the visible region will change upon obtaining hydrogen or an electron from the antioxidants. 

The DPPH assay is routinely used to evaluate the free radical scavenging potential of antioxidants based on the hydrogen atom transfer (HAT), or odd electron transfer (SET), which produce DPPH(H) or DPPH^−^, respectively [49]. Changes in color from violet to yellow as well as changes in the absorbance (515 nm) in the presence of an antioxidant indicate the degree of the radical scavenging potential of that compound.

Similar to DPPH, galvinoxyl is a rather stable phenoxy molecule. Because of its single electron, galvinoxyl exhibits a strong UV absorption band at 428 nm in ethanol solution. In the presence of antioxidants, galvinoxyl can be reduced irreversibly by accepting an electron or hydrogen radical to turn into a stable and diamagnetic molecule. In such cases, the original yellow color of galvinoxyl vanishes, regarding the number of electrons accepted [50]. Using this method, the antioxidant activity can be measured easily by the evaluation of absorption changes.

The ABTS radical is among the most abundant radicals for antioxidant capacity assaying [51]. This method is rapid, repeatable, and can be accomplished in both aqueous and organic solvents. The ABTS assay relies on the ABTS cation radical (ABTS˙+) generation in the presence of a strong oxidizing agent, such as hydrogen peroxide, potassium persulfate, or potassium permanganate. ABTS˙+ is more reactive than DPPH radicals. Usually, the relative ability of antioxidants for scavenging the (ABTS˙+) is compared with a Trolox (as a standard antioxidant). The changes of ABTS˙+ color in the presence of antioxidants as well as the suppression of ABTS˙+ characteristic absorption are the indicants of antioxidant potential [52,53]. The basis of the DMPD˙+ assay is the same as the ABTS^+^˙ assay. The maximum absorbance of DMPD˙+ is 505 nm [54]. 

## 4. Role of Antioxidant Materials in the Tissue Regeneration Process

### 4.1. Hard Tissues in the Craniofacial and Alveolar Area

#### 4.1.1. Craniofacial Bone Regeneration

Craniofacial fractures and bone defects in both the maxilla and mandible area occur as a result of trauma, congenital anomaly, and osseous deficiency, following resection of tumors, and subsequent tooth loss and extraction. Despite the significant ability of the bone to regenerate itself, external interventions are often essential for the comprehensive recovery of large bone defects and complex fractures. Oxidative stress and delayed angiogenesis are two important fundamental factors related to the low-quality healing process of large craniofacial defects [55]. Recently, the bone healing procedure has been enhanced with the application of chemical cues, such as biomaterials, biological factors, and stem cells. Due to the roles of ROS products in the healing progress of damaged bone tissue and their inhibitory effects on cell proliferation, the impact of antioxidant materials in combination with various scaffolds and medical hydrogels on osteogenesis and controlling ROS products is discussed here. In a study by Monte et al. [56], the amorphous silicon oxynitrophosphide (SiONPx), a kind of nano-antioxidant and a coating biomaterial, was investigated to enhance angiogenesis in craniofacial defects both in vitro and in vivo. SiONPx has the ability to increase antioxidants (SOD-1, catalase-1 [CAT-1]), angiogenic (CD31, ANG1) markers, and enhance vascular tubule formation while reducing ROS. During an in vitro study, human endothelial cells were exposed to ROS products, such as hydrogen peroxide, to stimulate oxidative conditions. They also conducted a study on rat standard-sized calvarial defects using plates coated with SiONPx. The results showed reduced cell death, enhanced microvascular networks, and the formation of a matrix by upregulating antioxidant and angiogenic factors, such as superoxide dismutase 1, nuclear factor erythroid 2–related factor 2 (Nrf2), and vascular endothelial growth factor A. The results of this study revealed that the atomic doping of phosphate into the nanoscale coating of SiONx could significantly increase the antioxidant capacity and angiogenesis of the coating to promote healing of craniofacial bone defects.

Natural antioxidants have been extensively applied to reduce ROS products at the damaged tissue site. For example, green tea is one of the most popular drinks, which mainly consists of tea polyphenols (catechins). The main chatechins found in green tea are epicatechin (EC), epigallocatechin (EGC), epicatechin-3-gallate (ECG), and epigallocatechin-3-gallate (EGCG). Among them, EGCG is much more abundant than other tea polyphenols. Several studies have revealed that EGCG has significant inhibitory impacts on abnormal processes, such as antioxidant, anti-inflammatory, anticancer, antifibrosis, and anti-collagenase effects. Regarding this, Rodriguez et al. [57] studied the impact of EGCG and α-tricalcium phosphate (α-TCP) on bone regeneration. These materials were applied after calvarial surgery in Wistar rats. They divided the rats into two groups. In the test group, the calvarial defect was filled with different doses of EGCG (0, 0.1, 0.2, 0.4 mg) combined with α-TCP particles, and, in the control group, the defect was not filled. The results revealed that the mixture of α-TCP with 0.2 mg of EGCG increased new bone formation in rat calvarial defects, and this mixture would become the potential bone graft material. Propolis is another natural antioxidant that is produced by honeybees from certain tree species. Caffeic acid phenethyl ester (CAPE) is an active component of propolis. It has been suggested that 10 μM of this substance could significantly inhibit the production of ROS in human neutrophils. Previous studies have also shown that CAPE has antioxidant, anti-inflammatory, and anticancer impacts [58,59]. In a study by Kazancioglu et al. [60], it was demonstrated that local application of CAPE at the level of 100 mmol/Kg and systemic application of it at 10 mmol/Kg/day significantly enhanced bone formation.

Dumanian et al. [61] evaluated a biocompatible scaffold, consisting of poly(polyethylene glycol citrate-co-N-isopropylacrylamide) (PPCN)/gelatin as a thermoresponsive material which has a low critical solution temperature. The scaffolds offered intrinsic antioxidant features due to PPCN’s ability to improve cell viability, chelate metal ions, and inhibit lipid peroxidation.

**Figure 2:**
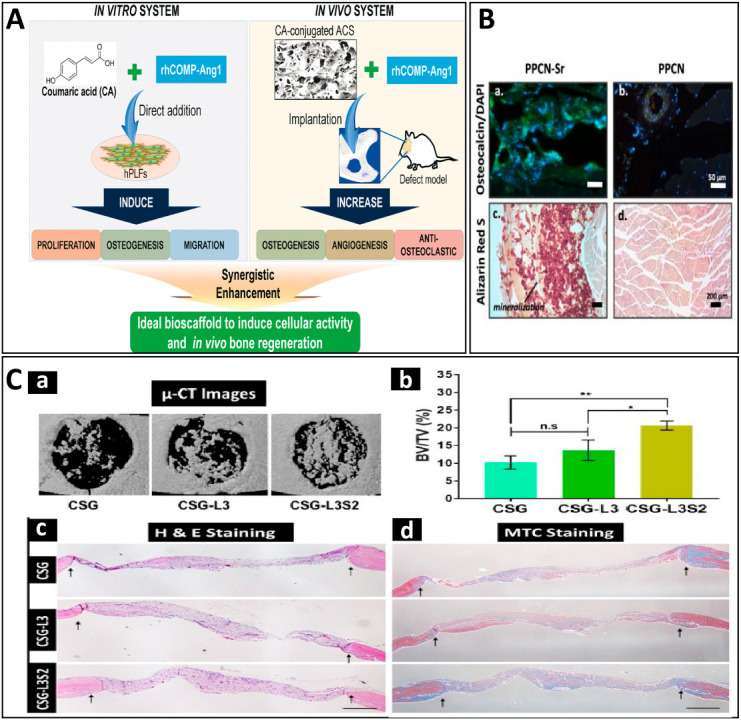
Application of coumaric acid and rhCOMP-Ang1 for the regeneration of hPLFs cells and critical-sized defects in rats (**A**) (Reprinted/adapted with permission from Ref. [63]. 2022, Elsevier). Application of PPCN-Sr revealed high levels of osteocalcin expression and cell infiltration in DAPI analysis in comparison to pure PPCN (**a**,**b**) Alizarin red stain test showed high mineralization in the PPCN-Sr group compared to the PPCN group(**c**,**d**) (**B**) (Reprinted/adapted with permission from Ref. [65]. 2018, Wiley Materials). Application of the chitin/poly(L-lactide-co-glycolide) (PLGA)-CaSO4 hydrogel with lactoferrin and substance P in calvarial bone regeneration in a critical-sized defect model in mice which was demonstrated with μ-CT images (**a**), quantification of B/TV% (**b**) (*: *p* < 0.05, **: *p* < 0.01, ns: not significant), H&E staining (**c**) and MTC staining (**d**) (black arrows indicate the mineralized and osteoid tissue) (**C**) (Reprinted/adapted with permission from Ref. [66]. 2021, Elsevier).

As popular natural sources, plant-derived phenolic compounds exhibit antioxidant, osteogenic, and anti-inflammatory features, which make them suitable for the improvement of bone regeneration [62]. In a study by Bahattarai et al. [63], coumaric acid (3-[4-hydroxyphenyl]-2-propenoic acid) was combined with collagen and recombinant-human cartilage oligomeric matrix protein-angiopoietin 1 (rhCOMP-Ang1) to enhance osteogenesis in mandibular critical-sized defects. The antioxidant features of coumaric acid caused scavenging of ROS and halted inflammation reactions. To summarize, this study showed that the synergetic interaction between rhCOMP-Ang1–enhanced angiogenesis and coumaric acid-related antioxidant responses could enhance the regeneration of critical-sized bone defects (Figure 2).

Resveratrol (RSV) is an anti-cancer and anti-inflammatory agent found in various plants, including grapes, berries, and peanuts. REV is also an antioxidant and pro-oxidant that was shown to have the ability to promote the osteogenesis of human adipose stem cells (hASCs) by upregulating RUNX2 gene expression. Regarding its application in bone regeneration, Wang et al. [64] fabricated scaffolds from collagen and RSV seeded with adipose stem cells to evaluate bone formation in the craniofacial region. The developed biocompatible scaffold could exhibit calcium deposits in hASCs, and improve osteogenic differentiation of hASCs, which is beneficial for craniofacial bone defects. Further studies on the role of antioxidant materials in craniofacial regeneration are summarized in Table 1.

#### 4.1.2. Alveolar Bone Regeneration

Periodontal tissue (periodontium) is a sophisticated and functional tooth-surrounding tissue. Periodontium includes both hard and soft tissue, such as alveolar bone, periodontal ligament (PDL), cementum, and gingiva [99]. One of the most inflammatory diseases in dentistry is periodontitis. Plaque and oral bacteria are the important causes of this disease. More than 50% of people who live in the world suffer from periodontitis. The main source of periodontitis is bacteria and their elements, which can cause inflammation and subsequently damage the periodontium. When periodontitis occurs, ROS are produced, mostly by hyperactive neutrophils, and may not be balanced by the host antioxidant defense system, causing tissue damage; therefore, over the last few years, in support of the treatment of periodontitis, various biomaterials have been utilized as a contact inhibition membrane in guided tissue regeneration (GTR), which is the current gold standard clinical regeneration treatment [100,101]. Herein, some available antioxidant materials involved in the regeneration of the hard part of periodontal tissue (alveolar bone) have been reviewed. Melatonin is a human hormone with various functions in human health, such as keeping humans safe from certain cancers, delaying senescence, stimulating type I collagen fiber formation, and improving bone formation. Due to the increasing levels of free radicals during periodontal diseases, the level of antioxidants, such as melatonin, will be raised in the saliva to protect the oral cavity against oxidative stress and regenerate alveolar bone and collagen fibers [102,103]. In addition, melatonin increases collagen IIIα1, decorin, and IL10; It also decreases the matrix metalloproteinase 1 (MMP1)/tissue inhibitor metalloproteinase protein 1 (TIMP1) ratio, which is suitable for alveolar bone formation in the oral cavity. Regarding this, Sola et al. [103] analyzed the antioxidant and osteogenic differentiation effects of melatonin on mesenchymal stem cells derived from the gingival mesenchymal stem cells (GMSCs). Results showed that melatonin protected GMSCs in a model of cellular damage (resulting from oxidative stress). Additionally, it increased alkaline phosphatase activity, enhanced bone formation ability, and upregulated RUNX2 gene expression. Similarly, the application of propolis in alveolar bone tissue regeneration was studied by Zohery et al. [104]. The effect of Egyptian propolis membrane on furcation defect regeneration in mongrel dogs was investigated. Overall, 24 class II furcation defects were created in mandibular premolars. The right-side defects were filled with nanohydroxyapatite and collagen membrane; the-left side defects were filled with propolis and collagen membrane. Histological evaluation after a 3-month follow-up revealed that propolis increased cellular proliferation and bone height compared to the nanohydroxyapatite group. Regarding the application of flavonoids as antioxidant agents, Arpornmaeklong et al. [105] studied the effect of chitosan/collagen hydrogels containing quercetin, a member of the flavonoid family, as a ROS scavenging matrix in stem cell delivery. The quercetin-bone γ-carboxyglutamate protein (BGP)-2:1 (*wt*/*wt*)-loaded chitosan/collagen hydrogels showed antioxidant properties and were able to improve the growth of hPDLSCs in a dose-dependent manner. Incorporating quercetin in the chitosan/collagen hydrogel promoted the hydrogel bioactivity that is useful for stem cell encapsulation. 

As a natural antioxidant source, curcumin, an active ingredient of turmeric, was studied in the literature. Curcumin is considered an effective material for decreasing inflammation and bone resorption. Zhou et al. [106] studied the effect of curcumin on bone after 30 days of daily administration of 100 mg/kg. They claimed that curcumin could reduce the inflammatory effect and alveolar bone loss in ligature-induced experimental periodontitis rats by reducing the levels of pro-inflammatory cytokines (and IL-6 and TNF-a) and osteoclastogenesis-related molecules, such as receptor activator of nuclear factor κ B (RANK) and receptor activator of nuclear factor kappa beta (RANKL). Another natural antioxidant material is Scutellaria baicalensis. Its extract contains baicalin and has shown anti-inflammatory and antioxidant properties. Chen et al. [107] carried out a study to investigate the impact of baicalein with different concentrations (1.25, 2.5, 5, and 10 μM) on hPDLCs proliferation. Their results demonstrated that baicalein increased the growth of hPDLCs, increased ALP activity, activated the Wnt/β-catenin pathway, and up-regulated the expression of viability markers, such as β-catenin and lymphoid enhancer factor 1 (LEF1). They found that baicalein can improve hPDLC differentiation into osteoblast via the Wnt/β-catenin pathway. A similar study provided by Kimura et al. [108] investigated the effect of baicalein on human cementoblast (HCEM) cells. Eventually, they found that baicalein was able to develop osteogenic differentiation of HCEM cells by the Wnt/beta-catenin signaling pathway, which makes it an effective candidate for periodontal tissue regeneration.

Inflammation and alveolar bone resorption are the common side effects after tooth extraction. Inflammation can increase amounts of osteoclast and pro-inflammation cytokines ((TNF)-α and interleukin 1 β (IL-1 β)), as well as RANKL and RANK. RANKL and RANK molecules are important for osteoclast activation, thereby increasing alveolar bone resorption. To address this issue, several biomaterials are applied as anti-inflammatory and antioxidant factors. For example, Kresnoadi et al. [109], combined polyethylene glycol (PEG) with demineralized freeze-dried bovine bone xenograft (DFDBBX) and xanthones (exists inside the mangosteen peel and containing dibenzopyran, an anti-inflammatory substance) in an animal study. Mangosteen peel extract (MPE) works as an anti-inflammatory and antioxidant agent. During this study, each mixture was applied locally after tooth extraction. One group received PEG only, the second group received DFDBBX + PEG, and the third group received MPE + DFDBBX + PEG. Finally, after 30 days, the immune histochemical evaluation demonstrated that the mixture of MPE with xenograft and PEG could improve the healing process and reduce bone resorption in tooth sockets. Hyaluronic acid (HA) also has distinctive physiochemical and biological properties, which make it beneficial in the treatment of the inflammatory process in medical fields such as periodontal disease treatment. It acts as an antioxidant agent by scavenging ROS and is able to stabilize the granulation tissue matrix. Moreover, it has a bone repair effect in periodontitis by inducing osteogenic substances, such as bone morphogenetic protein-2 and osteopontin. It was beneficial in controlling gingival bleeding and repair via decreasing peroxidase and lysozyme activities after a certain period. In the study by EI behaiy et al. [110], alveolar bone regeneration after tooth extraction in a canine model was performed by using a bioactive composite scaffold consisting of chitosan-bioactive glass (CH-MB) soaked in HA. During the experiments, the right extraction socket received bioactive composite (chitosan-bioactive glass (CH-MB)) scaffold soaked in 0.2% hyaluronic acid, while the left side received chitosan-bioactive glass scaffold alone. Hyaluronic acid-treated groups revealed significantly higher collagen content and crystal maturity compared to other groups after 6 weeks. 

**Figure 3:**
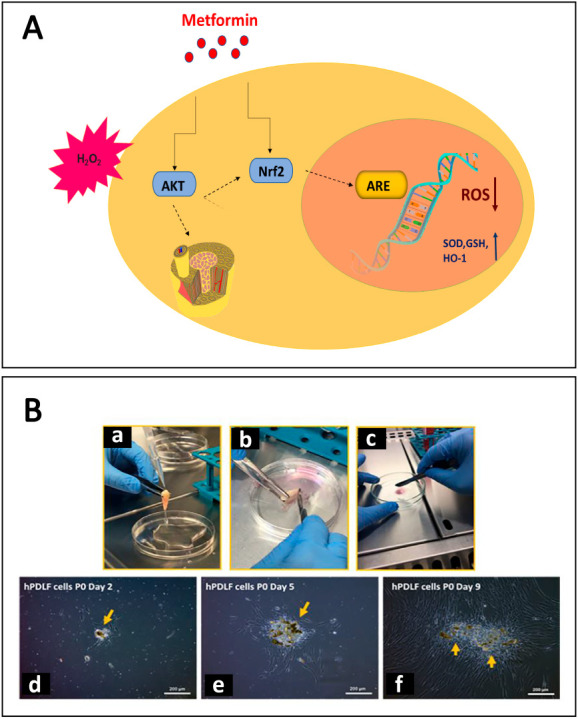
(**A**) The effect of metformin in promoting osteogenic differentiation and protecting against ROS through stimulation of the Akt/Nrf2 signaling pathway in cells, (**B**) Separation of human periodontal ligament fibroblast cells (hPDLFCs) (**a**–**c**), the effect of EPS on cell viability after 2, 5, and 9 days (**d**–**f**), yellow arrows indicate the cell colonies, (Reprinted/adapted with permission from Ref. [112] 2020, Elsevier).

Regarding the application of chemical drugs as antioxidants, Jia et al. [111] evaluated the effect of metformin as a common hypoglycemic drug on PDLSCs osteogenic differentiation. Application of 0.05 mM metformin for 72 h had no remarkable impact on decreasing the level of ROS and promoting the antioxidant capacity, but treatment after 7 days with 0.05 mM metformin had a significant effect on antioxidant capacity. Metformin was reported to stimulate Akt signaling and enhance the osteogenic differentiation of PDLSCs. In addition, metformin protects PDLSCs against H₂O₂-induced oxidative stress, partly via expressing Nrf2, which combines with the antioxidant response element (ARE) to increase the antioxidant enzymes or substances, including NQO1, HO-1, SOD, and GSH. The corresponded process can be observed in Figure 3. 

### 4.2. Dental Hard Tissue Regeneration

In the oral and maxillofacial regions, teeth are crucial for chewing food, speaking, and maintaining facial beauty. The teeth are composed of four parts: three kinds of hard tissues called enamel, dentine, and cementum; and dental pulp in the central zone [1]. Teeth have many similarities to bone in many aspects, including structural features, biomarkers, and mineralization activities; however, there are some differences. For example, while bone tissue undergoes continuous remodeling in life, including the development of new bones and the resorption of existing ones, dentine and cementum do not undergo remodeling; instead, they maintain their stability for a long time, while the dentine matrix is constantly deposited [113]. Many reasons exist for teeth to be damaged or lost, including caries, radiation, dental disease, trauma, tumors, genetic factors, and oral deficits caused by hepatobiliary diseases that induce tooth discoloration, enamel, and dentine hypoplasia [113,114]. Currently, dental issues are mainly treated by applying substances that have similar compositions, mechanical properties, or colors to teeth. Recently, bone tissue engineering has gradually drawn researchers’ attention to dental tissue regeneration. Here, we have reviewed antioxidant usage in dental hard tissue regeneration by the anatomical characteristics of the teeth [1].

#### 4.2.1. Enamel Regeneration

Enamel, the outer layer of the tooth crown, has the strongest bone structure in the body. A mature enamel consists of 95 wt.% carbonated hydroxyapatites, 4 wt.% water, and 1 wt.% organic matrix [115]. Dental caries is one of the most prevalent medical conditions worldwide, which continues to be a considerable public health challenge [116]. Dental plaque on teeth is a biofilm consisting of a smooth and sticky layer that often contains a gelatinous mass of bacteria. Natural antioxidants such as phenolic compounds can prevent radicals released from microbial activity from attaching to fluorescent compounds such as enamel. In addition, the effects of these materials as anticaries agents are significant even at low doses. These polyphenolic compounds found in herbs are believed to have many medicinal properties, including antioxidants, antibacterial, and anti-inflammatory effects [117]. Different polyphenol sources with anticaries properties have been identified. For example, tea, cranberries [118], and cacao bean husk extract [119] have been reported to have anti-Streptococcus mutans properties. Furthermore, polyphenols from Oenothera biennis, Paullina cupana, Sida urens L, Cistus incanus, Ziziphus jujuba, and Trachyspermum ammi all have inhibited S. mutants; thus, they can be used as dental caries preventatives [120]. 

**Figure 4:**
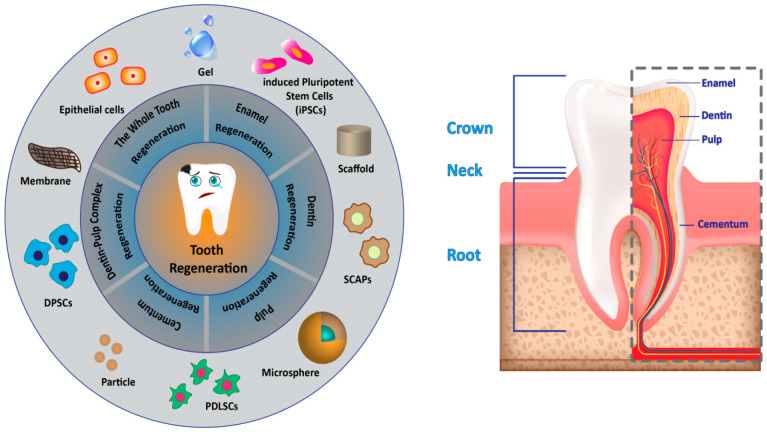
A graph showing tooth tissue regeneration based on cells and materials, including its structure.

Using a biofilm-demineralization model, Giacaman et al. [116] have reported that polyphenols in apple concentrate may exhibit anticaries activity even when sucrose is present and at very low concentrations. Additionally, Basir et al. [117] have demonstrated that curcumin has significant anticaries properties in low doses when exposed to sucrose. The enamel-forming cells, ameloblasts, and the stem cells, or enamel organs, are destroyed when teeth erupt; therefore, the engineering of the enamel tissue is a very challenging process. Different cell and material-based regeneration efforts for enamel regeneration have been reviewed in terms of the mechanism found by Olaru et al. [121] and Fu et al. [115] (Figure 4). However, more research is needed to learn how to regenerate enamel tissue and possibly use antioxidants to speed up enamel tissue engineering.

#### 4.2.2. Dentine Regeneration

Dentin, which is in the lateral walls of the pulp cavity and root canal and supports the enamel on its surface, constitutes the tooth’s main body [83]. Dentine is composed of 70% minerals, 20% organic matter, and 10% water [1]. Enamel caries that are left untreated will penetrate the deeper layer of dentin, break down the dentin collagen protein network, and cause severe pulpitis. Dentine collagen degradation may be facilitated by host-derived proteolytic enzymes, such as MMPs. Antioxidants can inhibit MMP activity and assist in the natural stabilization of collagen. For instance, hesperidin has shown anti-collagenolytic activity in a single-blind, split-mouth experiment [122]. When hesperidin was used to treat the samples, they were 24% more likely to protect the collagen than the controls. Most often, dentin regeneration is related to treating the dentin–pulp complex [121]. 

Dentinogenesis, the dentin formation process, begins when dental papilla cells (DPCs) differentiate into odontoblasts. Liu et al. [123] have investigated how EGCG, a component of green tea, affects the odontogenic differentiation of stem cells from apical papilla (SCAPs). In this study, EGCG promoted SCAP proliferation without altering SCAP migration at low concentrations (0.1 or 1 µM). By activating the BMP–Smad signaling pathway, EGCG enhanced the osteo-/odontogenic differentiation of SCAPs. Therefore, EGCG can be beneficial for the dentin regeneration process. In another study, Chen et al. [124] investigated the expression of SIRT4 during the differentiation of odontogenic DPCs and its potential mechanisms both in vivo and in vitro. Sirtuin SIRT4 was progressively upregulated during odontogenic differentiation, indicating that it is crucial for regulating odontogenic differentiation. The role of SIRT4 in dentin formation was demonstrated for the first time by modulating DPCs’ mitochondrial activity and antioxidant capacity. By incorporating these findings into further studies, it will be possible to understand how dentin regenerates and how SIRT4 regulates and functions during odontogenic differentiation. 

On the other hand, several studies are being done to make pulp capping materials more bioactive. For example, tideglusib, a regenerative drug with antioxidant and anti-inflammatory properties used for Alzheimer’s, has also shown a treatment role for dental cavities. Rao et al. [125] synthesized tideglusib-loaded bioactive glass nanoparticles (tideglusib-BgNPs) and incorporated them into calcium silicate cement at different concentrations. By adding tideglusib-BgNPs to calcium silicate cement, the cytotoxicity of calcium silicate cement was lessened, and cell growth and migration were improved, leading to the better treatment. As a result, tideglusib-BgNPs have the potential to be utilized in pulp therapy, in which new dentin is formed rapidly, resulting in faster dentin healing and repair. In addition, as demonstrated by Minamikawa et al. [126], the amino acid derivative N-acetyl cysteine (NAC) was able to decrease cytotoxicity and increase the conductivity of mineralized tissues in the common dental restoration material resin-modified glass ionomer (RMGI). Upon NAC supplementation, the percentage of viable cells, gene and protein expression levels related to odontoblasts adhesion/spreading were significantly increased. Enhanced behaviors in dental pulp cells with NAC supplementation were related to reduced intracellular ROS generation and elevated intracellular glutathione reserves. These findings offer new perspectives for developing dental restorative materials capable of regenerating dentin-like mineralized tissues.

#### 4.2.3. Cementum Regeneration

Cementum is a mineralized bone-like tissue created by cementoblasts that surround the tooth root and connect the tooth by PDL to the alveolar bone [127]. Therefore, it has a crucial role in the regeneration of periodontal tissues. The regeneration of periodontal tissues, especially cementum, is crucial to periodontal health. In a study by Gauthier et al. [128], the effects of common osteogenic stimulants and vitamin C on the expression of cementogenic genes were investigated. Their results showed that periodontal ligament stem cells (PDLSCs) could not be guided toward cementogenic lineages by a common osteogenic medium containing vitamin D3, Dex, and β-glycerophosphate, indicating that, in response to such stimuli, pathways promoting specific cementogenic genes are inhibited rather than induced. In contrast, vitamin C was able to increase cementogenic gene expression. In addition, transplanting PDLSCs treated with vitamin C into immunocompromised mice in this study resulted in considerably more ectopic cementum and bone formation. Thus, for clinical cell-based periodontal regeneration, vitamin C treatment alone can be feasible and safe. In another study, Leon et al. [129] suggested that interleukin-11, a cytokine with a critical function in bone metabolism can boost osteoblast differentiation markers, including osteocalcin, Runx2, and bone sialoprotein expression in periodontal ligament cells treated by vitamin C, synergistically. These results may contribute to the regeneration of cementum, since the expression of the bone sialoprotein gene in newly formed osteoblasts is known to be induced by cytokines and hormones and is associated with de novo cementum and bone mineralization. Salmon et al. [127] have performed proteomic research on human dental cementum, which contributes to developing more effective and predictable periodontal regenerative therapies. They found that dental cementum and related cells in mouse, pig, and human teeth contain superoxide dismutase 3 (SOD3). SOD3 is one of three oxidoreductases involved in protecting cells from O_2_^−^ and peroxynitrite formed during aerobic cellular respiration and oxidative stress. This result indicated the crucial role of SOD3 as an endogenous antioxidant in defending cementum against oxidative stresses during cementum formation or maintenance.

### 4.3. Oral Soft Tissue Regeneration 

#### 4.3.1. Periodontal Soft Tissue Regeneration

Periodontitis is a very usual infectious disease in the oral cavity and leads to the destruction of the periodontium. As mentioned previously, the periodontium consists of hard tissue (alveolar bone and cementum) and soft tissue (gingiva and periodontal ligament).

Naturally derived biomaterials with multifunctional properties have been suggested to have astonishing effects on periodontal repair and regeneration. Natural microbial exopolysaccharides (EPS) have attracted scientists’ attention because of their potential pharmaceutical properties such as antioxidant, anticancer, and anti-inflammatory effects. In a study by Kibar et al. [112], EPS (50 mg/mL) exhibited 70% inhibition on biofilm formation, which could be in favor of suppressing periodontitis. In addition, dose-dependent antioxidant effects were detected for these biomaterials. Moreover, EPS increased the viability of hPDLFCs. Consequently, they concluded that EPS in the form of powder could enhance periodontium soft tissue regeneration and protect against free radicals. Another study related to the soft part of the periodontium was conducted by Pradeep et al. [130] to investigate the effect of a well-known natural material, aloe vera (AV), on periodontal diseases. Having antioxidant, anti-inflammatory, healing-promoting, antimicrobial, hypoglycemic, and immune-boosting properties, AV has been used in medicine and dentistry for several years. The results of Pradeep’s study revealed that AV gel could enhance the plaque index and probing depth in patients. The antioxidant function of the enamel matrix derivative (EMD) in the initial stage of periodontal regeneration was analyzed in a study by Takeda et al. [131]. They designed an animal study by resembling oxidative stress conditions via the induction of hyperglycemia. Eventually, EMD enhanced the initial stage of wound healing in periodontium by suppressing of hyperglycemia-induced oxidative stress. In addition, a higher amount of connective tissue formation was observed by the application of EMD. Table 1 summarizes the reported work on the effect of antioxidant materials on periodontal soft tissue regeneration [74,75,76,77,78,79,80,81,82,83].

#### 4.3.2. Oral Wound Healing

Oral wound healing is a complicated biological process aimed at the restoration of anatomic structure and function, achieved by four different phases such as inflammation, hemostasis, proliferation, and remodeling [132]. Non-healing oral wounds can significantly increase morbidity and possess negative effects on patients due to the unique properties of the oral cavity. During wound healing, excessive oxidative stress hinders the process by inducing inflammatory reactions. In addition, ROS suppress the regeneration of wound tissue by inhibiting the functions of endogenous stem cells and macrophages. Many researchers have reported the application of ROS-scavenging materials in the healing of oral wounds to speed up the regeneration process. Regarding this, Gu et al. [133] investigated vitamin B2-modified iron oxide nanoparticles for promoting oral ulcer healing. Although oral ulcers are harmless and self-healing, they can be painful and reduce the patient’s ability to eat, drink, brush, and even speak, affecting his quality of life and working efficiency. Usually, oral ulcers are associated with inflammation and high levels of ROS, which exacerbate the patient’s symptoms. It was reported that vitamin B2-modified iron oxide nanoparticles improved peroxidase-like, catalase-like, and superoxide dismutase (SOD)-like activities. Additionally, vitamin B2 modification significantly increased the ROS-scavenging ability and protected human oral keratinocytes (HOK) and BALB/3T3 cells from hydrogen peroxide (H_2_O_2_).

Tooth extraction is a daily procedure in every dental office that results in hard and soft tissue damage. In addition, a great number of factors in the oral cavity, including various microorganisms, saliva, and prosthetic appliance, can remarkably disrupt the process of wound healing after tooth extraction and surgery. Complications that happen following a tooth extraction are inflammation, swelling, and pain, which can notably alter the patient’s quality of life. As a result, facilitating wound healing after a tooth extraction is very crucial in dentistry. During the process of wound healing, the production of ROS can play a critical role in the fight against bacteria. Hence, locally applied antioxidants can provoke tissue regeneration by minimizing inflammation. Additionally, they can keep the wound safe against bacterial infection and improve cellular proliferation [132]. Regarding the application of natural antioxidants in accelerating the healing process in post-extraction wounds, Krismaya et al. [134] used flavonoids, tannins, and saponins found in lime peel (approximately 50% lime peel in the post-extraction socket in the treatment group) topically as an antioxidant and antibacterial agent to promote fibroblast proliferation and angiogenesis in the tooth socket after extraction. They used 24 Wistar rats and divided them into case and control groups. After 5 days of application, an increase in blood vessel formation and fibroblast growth was observed. In another study conducted by Fidoski et al. [135], the local effects of nano-emulsion gel containing propolis, vitamin C, and vitamin E on wound healing were investigated. The nano-emulsion gel was applied three times a day via a thin film to cover the wound with a gentle massage for 1 min. Furthermore, local application of this nano gel especially in the first three days after surgery, was able to enhance oral wound healing and also provide suitable protection against early surgical complications such as inflammation. 

### 4.4. Dental Pulp Tissue

**Figure 5:**
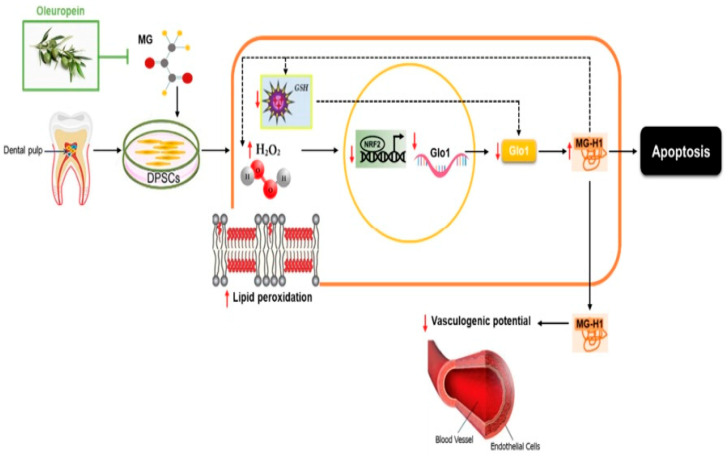
Mechanism of action of MG and OP on DPSCs. MG would provoke oxidative stress as a result of the Nrf2 pathway and down-stream Glo1, with the consequent release of MG-H1, eventually leading to DPSC apoptosis, while OP, by preventing this mechanism, plays a pivotal protective role against MG damage in DPSCs (Reprinted/adapted with permission from Ref. [138] 2021, MDPI).

In dental pulp tissue, similar to other tissues in the human body, medical treatments such as the removal of tissue during restorative and endodontic treatments or tooth movement during orthodontics, can induce oxidative stress and eventually increase the levels of pro-inflammatory factors, including TNF-α and interleukin-1β. Therefore, high levels of oxidative stress could be alleviated by applying antioxidants, which may enhance the healing of dental pulp tissue [136]. In dental pulp tissue, ROS stress could damage different organic molecules, including proteins and DNA. Therefore, the production of internal antioxidants or the application of various biomaterials as an antioxidant treatment is helpful to neutralize the harmful effects of ROS in the pulp tissue [137]. Up to now, several studies have been conducted to study the role of antioxidant biomaterials in enhancing dental pulp tissue regeneration and suppressing ROS effects. Monache et al. [138] evaluated the role of oleuropein (OP) (a phenolic compound found in olive fruit and leaves with antioxidant and anti-inflammatory properties) to prevent (methylglyoxal) MG-dependent glycative stress in human dental pulp stem cells (DPSCs). MG is a potent forerunner of glycation stress, which can cause several diseases, such as oral disorders. In this study, human dental pulp stem cells were exposed to the OP (50 µM) for 24 h before the application of MG (300 µM) for an extra 24 h. They found that OP was able to prevent MG-induced glycation stress and DPSCs destruction (Figure 5). Their research opened a new approach to the prevention of MG-related oral diseases. In another study, Bagheri et al. [139] investigated the antioxidant and anti-inflammatory impact of quercetin in dental pulp tissue among diabetic rats. Rats were gavaged daily with quercetin (25 mg/kg) for forty days. Their results showed that quercetin increased the number of internal antioxidants such as SOD1, CAT, and GPX1 molecules against ROS products and enhanced dental pulp tissue regeneration.

Cinnamaldehyde (CA), an important biological substance isolated from the stem bark of Cinnamomum cassia, has various biological functions, such as anti-inflammatory, anti-cancer, antibacterial, and antioxidant features. CA can act as a stimulator of Nrf2, a main regulator of the cellular antioxidant defense. Regarding this, in a study by Kim et al. [140], HDPSCs were treated with CA at 20 μM for 0.5, 1, 3, 6, or 24 h. They observed that CA has a cytoprotective impact against H_2_O_2_-induced oxidative stress in hDPCs by increasing the level of HO-1 via the Nrf2 signaling pathway. They concluded that CA may provide strong protection for dental pulp stem cells against free radicals.

Dentin-pulp regeneration following tooth decay treatment plays an essential role in ensuring tooth viability. When hDPSCs differentiate into odontoblasts, intracellular ROS levels increase, consequently reducing the differentiation potential. Furthermore, antioxidants play a crucial role in odontoblast differentiation media, so preconditioning stem cells with antioxidants will synergistically enhance their differentiation. Researchers [92] have incorporated an insoluble inorganic ceria nanoparticle (CNP), an antioxidant used for tissue regeneration in hard or nervous systems, into mineral trioxide aggregate (MTA) to accelerate odontoblast differentiation by reducing ROS levels. As a result of the utilization of CNP-incorporated MTA (CMTA), odontoblastic differentiation was accelerated without compromising compression strength. In this way, CMTA may provide dental materials capable of regenerating dentin-pulp complexes by directing pathological intracellular ROS toward beneficial biological outcomes. Researchers [141] have evaluated the impact of grape seed extract (GSE), an antioxidant polyphenol, on the growth and mineralization of undifferentiated pulp cells (OD-21) and odontoblast-like cells (MDPC-23). MDPC-23 cells showed increased proliferation and protein content after 7 and 10 days, respectively. Additionally, undifferentiated pulp cells and odontoblast-like cells showed higher ALP staining intensity after 7 and 10 days. In this study, GSE enhanced differentiated cells’ functional activity more than OD-21 cells. However, to confirm GSE’s benefits for dentin regeneration, more studies should be conducted at various concentrations of GSE. 

One factor that can disturb dental pulp cells is resin (co)monomer (particularly 2-hydroxyethylmethacrylate (HEMA)). It is separated from restorative dental materials during dental treatment and diffuses into the dentin micro-channel and pulp-causing bloodstream and could affect the cells’ integrity. To eliminate the inflammatory effect of HEMA, application of antioxidant materials with HEMA is recommended. For example, Diomede et al. [142] evaluated the effect of ascorbic acid (AS), an antioxidant and anti-inflammatory molecule, on the inflammatory status induced by HEMA in hDPSCs. The cells were induced with 2 mM HEMA and then treated with 50 µg/mL AS locally. Overall results showed a reduced level of pro-inflammatory markers, increased cell proliferation, and promoted downregulation in ROS products. 

### 4.5. Cartilage Tissue

**Figure 6:**
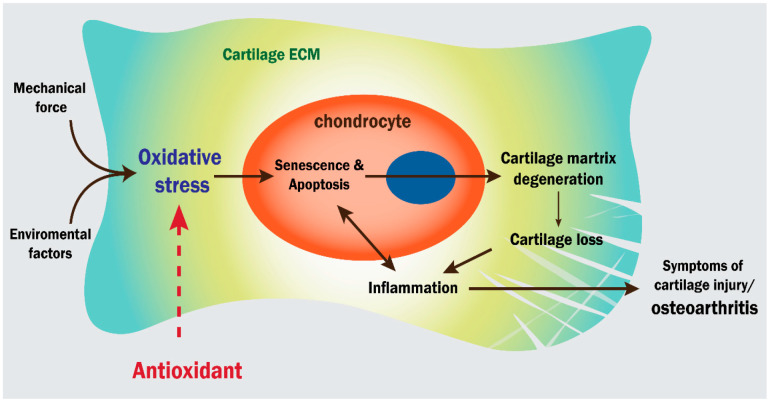
Illustration of oxidative stress’ role in cartilage degeneration and osteoarthritis. Antioxidants interfere with this pathway, as indicated by the red arrow.

There is a limited ability for cartilage to regenerate itself [143]. Degeneration of cartilage can be associated with ROS signaling, which is aggravated by inflammation, as seen in osteoarthritis (OA). Since cartilage lacks a vascular system, chondrocytes are dormant under normal circumstances and inhabit a hypoxic condition. A mitochondrial dysfunction triggers the activation of chondrocytes, increasing ROS production and resulting in more OA. Additionally, according to numerous studies, age can exacerbate oxidative stress, resulting in a loss of balance between ROS generation and antioxidative responses [144]. Several antioxidants and free radical scavengers have been demonstrated to delay cartilage degradation (Figure 6), including flavonoids (curcumin, quercetin, resveratrol), phenols (vitamin E), ubiquitin, thiols (thioredoxin and glutathione) [145,146], chondroitin sulfate (CS), xanthan gum [147], dopamine melanin [148], alginate, hyaluronic acid [147,149], fullerene and fullerol [150], etc. Through the use of these materials, antioxidative substances, such as glutathione, catalase, nuclear factor erythroid-2-related factor 2 (Nrf2), and superoxide dismutase, can be increased and resulting in downregulating of ROS [144].

Researchers have extensively studied the antioxidant properties of black tea polyphenols, named theaflavins (TFs) [151]. For example, Li et al. [152] investigated the protective impacts of TFs on chondrocytes and discovered a reduction in ROS levels and apoptosis rates. CS is also an anti-inflammatory glycosaminoglycan found in soft tissues. Woo et al. [153] have found that skate cartilage CS reduced inflammatory cytokine secretion and ROS levels in the murine cell line RAW 264.7 that had been treated with lipopolysaccharide.

Even though antioxidants can be administered systemically, they do not appear to be retained at the site of damage; therefore, their local delivery can be beneficial. Liang et al. [154] investigated the antioxidative potential of PCL-grafted lignin in rabbit OA models and H_2_O_2_-stimulated human chondrocytes, which indicated an autophagic mechanism. Likewise, poly(lactic acid)(PLA)-lignin nanofibers were tested by Liang et al. [155] as antioxidant biomaterials for cartilage regeneration. In this study, PLA was grafted into lignin using ring-opening polymerization and electrospun into nanofibers. The results of in vitro studies indicated that PLA-lignin nanofibrils could promote the differentiation of chondrogenic stem cells and protect them from oxidative stress. Additionally, lignin nanofibers were found to enhance cartilage regeneration within six weeks of their implantation. The antioxidant properties of alpha-tocopheryl succinate (α-TOS) and TNF-α siRNA co-loaded poly(amidoamine) dendrimer-entrapped gold nanoparticles (Au DENPs) were studied by Lie et al. [156]. Thanks to the utilization of α-TOS for this study, the macrophage antioxidant capacity was enhanced, and in vivo studies in the OA model confirmed the downregulation of inflammatory cytokines. In addition, in a study by Pei et al. [150], polyhydroxylated fullerene C60 (fullerol) nanoparticles were used as ROS scavengers, which caused a reduction in the production of NO and expression of proinflammatory genes.

A silk/graphene oxide-based meniscal scaffold coated with tannic acid/strontium was designed to reduce ROS and regulate inflammation in an OA model [157]. This scaffold significantly reduced inflammatory factor (interleukins 6 and 8, and MMPs) expressions and enhanced ROS scavenging abilities in the knee tissues of rats. The incorporation of ionic liquids (IL) into bacterial nanocellulose membranes (BC) [158] has also shown anti-inflammatory and antioxidant properties. In another attempt to treat OA, Xue et al. [159] constructed metal-organic frameworks (MOFs) that decorated mesoporous polydopamine nanoparticles and conjugated these nanoparticles with collagen-II-targeting peptides. The MOF shells and pores were loaded with bilirubin and rapamycin, respectively, to modify the inflammatory response and trigger autophagy. These nanoparticles reduced cartilage degeneration, controlled inflammation, and protected chondrocytes.

Considering that after cartilage degeneration, inflammatory factors such as interleukin-1 (IL-1), IL-1β, IL-6, TNF-α, and ROS species are widely expressed and contribute to the inflammatory response, a hybrid scaffold was developed by Wu et al. using PLGA filled with a hyaluronic acid methacrylate (HAMA) hydrogel containing ROS-sensitive hyper branched polymers that pose thioketal linkages to scavenge ROS [160]. Aside from modulating ROS, the scaffold was able to reduce inflammation and promote hyaline cartilage regeneration.

Several examples of antioxidant utilization in cartilage tissue engineering were discussed above. However, it would be interesting to investigate the effectiveness of these antioxidants in the regeneration of oral and maxillofacial cartilage tissue. In the oral and craniomaxillofacial regions, cartilage regeneration includes TMJ cartilage, auricular cartilage, and nasal cartilage regeneration [1].

#### 4.5.1. TMJ Cartilage

Craniofacial skeletal defects primarily affect bony structures, whereas chondral and osteochondral deformities are less common but have serious consequences. TMJ tissue in adults contains articular cartilage [161]. As a critical joint, TMJ maintains mandibular movements, including opening and closing the mouth. The TMJ is a ginglymoarthrodial joint consisting of a bone-cartilage interface and a bilateral synovial disc, whose function is heavily reliant upon the disc [1]. Even with the rarity of cartilage defects, TMJ problems have significant clinical implications in maxillofacial surgery. TMJ structures are commonly degenerated or lost due to trauma, infection, or autoimmune disorders. In general, TMJ disorders are classified into arthritic, growth, and non-arthritic disorders. The first class is caused by inflammation and appears in patients with pain and facial deformities; treatment is aimed at removing risk factors and inflammatory markers. The second type appears in facial deformities, and treatment is aimed at removing the tumor by surgery. In the third type, mechanical derangement can play an important role. Some common clinical complaints in this type are luxation and disc replacement. It is important to reduce mechanical obstruction when treating this type of disorder. Consequently, treating TMJ defects can be challenging, depending on the causes of the defect [161,162]. Hence, researchers prefer to use natural, synthetic, and bio-compatible materials, such as antioxidants, in joint treatments to repair joint disorders [163]. For healing TMJ disorders, some researchers and clinicians have tried to fabricate different prothesis to improve the oral cavity function. Regarding this, Pagano et al. [163] evaluated the effect of different materials, such as PMMA resin (for making prosthesis), on the morphology, function, proliferation, and viability of the human keratinocyte. These cells have a critical role in creating a biological seal between the prosthesis and connective tissue that favors prosthesis adaptation to supporting tissues during joint treatments. Resin materials could induce p53 expression in human keratinocytes, preventing cell apoptosis and promoting cell viability against various damages in the oral cavity. 

In a narrative review by Braz et al. [164], oxidative stress (im)balance and its potential contribution to temporomandibular disorders (TMDs) were evaluated using synovial fluid. It was reported that oxidative damage and the severity of intra-articular TMD were positively correlated. According to the results, oxidative stress causes intra-articular damage by both introducing reactive oxygen/nitrogen species and reducing antioxidant defense. Hence, there is a strong connection between inflammation of the TMJ and oxidative stress. Research by Ueno et al. [165] investigated the effects of NAC on oxidative damage to TMJ chondrocytes isolated from condylar bone surfaces. NAC, a glutathione derivative, can promote glutathione redox cycles within cells as well as scavenge ROS. This study demonstrated that NAC restored intracellular glutathione reserves and reduced intracellular ROX induced by H_2_O_2_. In another study, NAC inhibited apoptosis in chondrocytes stimulated by nitric oxide (NO) [166]. Accordingly, due to the role of NAC in preventing cell death and the impairment of TMJ chondrocyte function related to oxidative stress, it deserves further investigation to determine its efficiency in vivo.

TMJ osteoarthritis (TMJOA), a multifactorial disease of the TMJ primarily caused by inflammation, can affect people of any age [167]. TMJOA involves non-inflammatory and inflammatory changes and can affect all TMJ tissues and cause anatomical abnormalities. This disorder causes persistent inflammation of synovial tissues, cartilage degeneration, and subchondral bone remodeling. Despite several risk factors contributing to TMJOA, no clear pathogenesis has been identified. Often, TMJOA occurs in patients who suffer from TMDs [168]. 

There are currently some therapies for TMJOA, such as hyaluronic acid, that can lubricate joints. However, injecting hyaluronic acid during acute inflammation is not recommended. It is therefore urgently necessary to discover new molecules and strategies to slow and prevent the progression of TMJOA [167,169]. IL-1β triggers a range of molecular interactions that lead to oxidative stress and TMJ inflammation. Therefore, in a study by Jiang et al. [167], IL-1β was used as a pro-inflammatory substance. They investigated curcumin’s anti-inflammatory and antioxidant properties in TMJOA. The results of this study showed that curcumin treatment prevented the expression of COX-2, IL-6, iNOS, and MMPs (i.e., MMP-1, MMP-3, etc.) in human chondrocytes from TMJ cartilage samples. It also increased the mRNA expression of the cartilage-building factors COL2A1 and ACAN and reduced the damage caused by IL-1β-induced oxidative stress. Pathway analysis showed curcumin stimulates the Nrf2/ARE pathway in TMJ inflammatory chondrocytes. These effects were notably reversed by specific Nrf2 siRNA. 

In light of the positive link between inflammation and COX-2 expression in OA, Li et al. [170] investigated RSV as an antioxidant to prevent mandibular condylar cartilage (MCC) degradation. In the pathogenesis of TMJOA, NF-κB is a critical transcription factor of immune and inflammatory responses. In this study, TMJOA was generated in mice models by injecting collagenase. Then, RSV (100 μg/10 μL) was injected three times a week for 4 weeks in the treated group. Results showed that inflammation could lead to the degradation of MCC both in laboratory conditions and in vivo, but RSV was capable of reversing it. As a result, RSV downregulated the expression of COX-2/NF-κB/MMP and induced cartilage markers. According to the results, RSV was able to inhibit chondrocyte apoptosis in TMJOA by exerting antioxidant actions and downregulating the COX-2/NF-κB pathway. 

**Figure 7:**
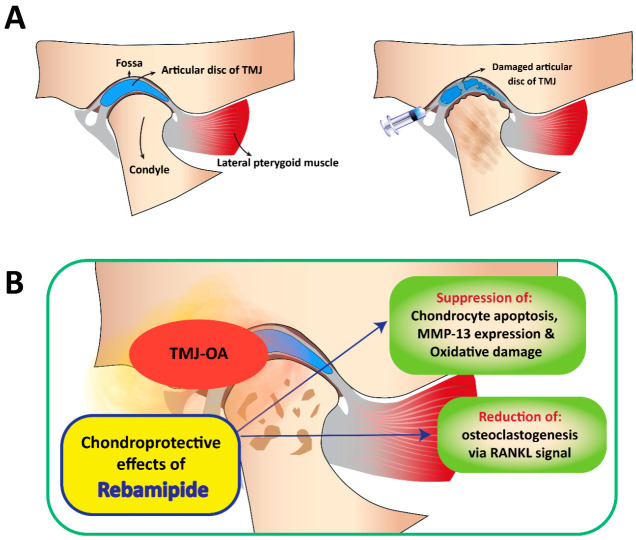
(**A**) A schematic showing TMJ anatomy and the commonly targeted areas to address TMD. Injectable biomolecules can be delivered intra-articularly through a syringe with a needle to treat TMD through the TMJ capsule. (**B**) The rebamipide chondroprotective effect in TMJOA.

Izawa et al. [168] have studied the antioxidative properties of rebamipide in a review article. Rebamipide, a quinolone derivative, has been used for gastric ulcer treatment, which has also demonstrated antibacterial and anti-inflammatory properties. Additionally, studies have shown that rebamipide suppresses damages caused by oxidants and promotes extracellular matrix homeostasis in articular chondrocytes, which attenuates cartilage degeneration (Figure 7). Rebamipide’s ROS-scavenging property may mediate its chondroprotective effects on cartilage affected by TMJOA. In addition, rebamipide reduced osteoclast differentiation by stimulating mitogen-activated protein kinases. As well, it may be possible that the chondroprotective effects of rebamipide are associated with its ROS-scavenging capabilities. Together, these results suggest that rebamipide reduces osteoclastogenesis and cartilage destruction in TMJOA. Although studies for TMJ regeneration are emerging, the difficulty in determining pathogenesis mechanisms, reproducing anatomical structures accurately, and finding appropriate animal models can limit research. It will be possible to apply antioxidants in this area more effectively in the future if a pathogenic mechanism can be identified and more research is performed.

#### 4.5.2. Auricular and Nasal Cartilage Regeneration

Auricular and nasal cartilage have an important role in maintaining their appearance and functionality. The nasal and auricular cartilage differ from the articular cartilage in the TMJ in terms of their structure and function [1]. Auricular cartilage is elastic cartilage composed of a collagen type-II network and sulfated glycosaminoglycan. It does not self-repair or regenerate despite its high mechanical strength. Several auricular deformities can develop as a result of congenital defects, burns, and trauma, for which tissue engineering has been suggested as the most beneficial treatment [171,172].

An artificial auricular cartilage based on silk fibroin (SF)/polyvinyl alcohol (PVA) was developed by Lee et al. [171]. This 3D cartilage with an ear shape was formed in rats after subcutaneous implantation of the chondrocyte-seeded hydrogel. Histological analysis confirmed the existence of mature cartilage with typical lacunar morphology, in vitro and in vivo. This 3D scaffold can be a basis for using antioxidants in auricular cartilage regeneration.

The condition of microtia, one of the ear disorders, refers to deformities of the external ear (auricle and pinna) caused by congenital birth defects. A tissue-engineered scaffold of ear pinna cartilage has been evaluated using various chemical treatment techniques to create a flexible and human-sized ear. In this study [173], 5% Dimethyl sulfoxide (DMSO) (*v*/*v*) was utilized as an antioxidant treatment in one of the decellularization methods followed by freeze-thaw cycles. This method produced more durable pinna-shaped cartilage that was mechanically more stable and effective at removing antigenic material.

Relapsing polychondritis (RP) is another disorder in which systemic cartilage tissues, such as the external auricle, nose, respiratory tract, and joints, are inflamed. Qi et al. [174] investigated how curcumin might exert its chondroprotective effects on H_2_O_2_-treated primary chondrocytes in vitro and discovered that curcumin and H_2_O_2_ co-treatment significantly reduced growth inhibition. In addition, curcumin inhibited the production of inflammatory mediators.

Antioxidants of different types have also been tested for their effectiveness on reimplanted auricular composite graft survival [175]. The researchers discovered that dimethyl thiourea (DMTU), hyperbaric oxygen (HBO), and melatonin, all resulted in significant improvements in auricular composite graft survival after seven days. Nevertheless, on day 21, the average survival rate in all groups had reached 13% to 14%. In the first postoperative week, HBO had the worst effect on survival, while DMTU had the best. Both DMTU, an oxygen-free radical scavenger, and HBO have been shown in animal and human studies to improve tissue flap survival. Melatonin acts similarly as an oxygen radical scavenger in many animal models, protecting tissues from oxidative stress. There are currently very few studies on the utilization of antioxidants in the regeneration of nasal cartilage. In a research study performed by Çakan et al. [176], topical application of curcumin was found to improve epithelial and cartilage regeneration upon nasal septal perforation. It was demonstrated that the curcumin group had higher amounts of collagen and granulated tissue. Further research will be required in the area of nasal regeneration and the use of antioxidants to regenerate cartilage tissue in the nasal area.

### 4.6. Nerve Tissue Regeneration

Facial muscles are exceptionally functional for humans, have aesthetic functions, and have an important role in facial expression movements. The facial or seventh cranial nerve is the main nerve that controls all muscles that contributed to facial expression [177]. Facial nerve paralysis is the most common neural disorder in the cranium, and it may be caused by several reasons [177]. After nerve paralysis, trauma is the second most common type of neural disease in the cranium. Following post-traumatic recovery, inflammation makes extensive fibroconnective tissue and scars on the nerve tissue, and consequently, neuron tissue may lose its function [178]. Nervous system injuries should be reduced to maximize nerve regeneration. Oxidative stress and greater numbers of oxygen-derived free radicals, including fatty acids and arachidonic acids from cell membranes, are considered to be harmful to the neuronal cells after axotomy. In this regard, antioxidants could be used as a strong defense system to remarkably combat the oxidative damage induced by nerve injuries [178]. 

One of the short-lived, free radical gases in the central nervous system (CNS) is NO. NO may disrupt mitochondrial metabolism and induce apoptosis, and it acts as a neurotoxic agent during brain ischemia and hypoxia. NO is a toxic agent for cells and produces other deleterious free radicals in the nervous system. For this reason, Japanese scientists have discovered an influential antioxidant and free radical scavenger which increases the activity of the mitochondrial fraction SOD, which is called Tokishakuyakusan (TJ-23). In a study by Ito et al. [179], the antioxidant impact of TJ-23 (0.53% *w*/*w*) on the NO synthesis pathway in the facial motor nucleus (FMN) motoneurons after peripheral axotomy was assessed by the NADPH-d histochemistry method in 40 rats. After 56 days, the number of remaining alive motoneurons in the ipsilateral FMN was notably greater in the TJ-23 rat group than in the non-treated group. The scavenging effect of TJ-23 against NO indicates that this herbal agent is a beneficial protective material for damaged neural cells following axotomy. Moreover, TJ-23 might hinder neuronal cell damage because of its scavenging activity against NO and could finally keep FMN safe.

Werdnig–Hoffmann disease is a neuropathic disease in humans with genetic causes and causes lower motoneuron degeneration with associated skeletal muscle atrophy. It has been observed that ROS may cause the pathogenesis of this disease. To investigate the relationship of this illness with ROS activity, Henderson et al. [180] have evaluated the effects of NAC on neuronal regeneration in wobbler mice (1% solution of the glutathione precursor NAC) after 9 weeks. As a result, a remarkable reduction in motor neuron loss, increased glutathione peroxidase levels with the increased axon caliber in the facial nerve, and increased muscle mass in the facial area were observed. The effects of oral administration of NAC may be appropriate in recovering neuronal loss disorders. In addition, in a study by Hoshida et al. [181], the effects of vitamin E as an antioxidant substance after nerve injury were assessed. Their study consisted of three groups of mice that received 200 mg/mL (high dose) and 20 mg/mL (moderate dose) of vitamin E, as well as a control group. After 4 weeks of nerve avulsion, the number of surviving motor neurons in the ipsilateral FMN was remarkably more significant among vitamin E-treated rats in comparison to the control group. 

**Figure 8:**
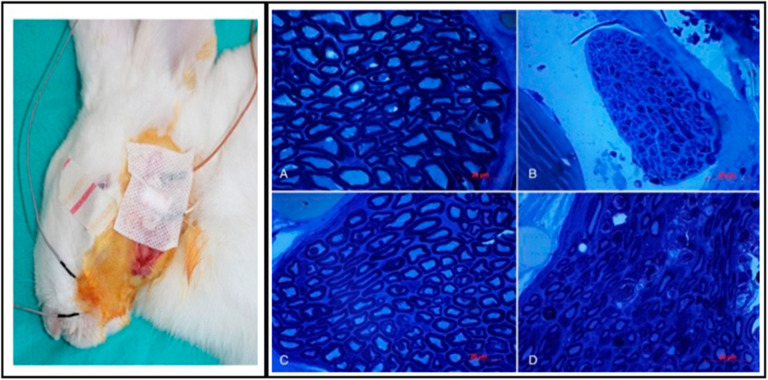
Electrophysiological and histological evaluation of the rabbit facial nerve in the control group (**A**), degenerated neural tissue (**B**), thymoquinone-treated group (**C**), and methylprednisolone-treated group (**D**) (Reprinted/adapted with permission from Ref. [183]. 2016, Elsevier).

Recently, some studies have applied PEG fusion protocols in neural regeneration. During this protocol, the neurotmesis of some facial branches is immersed in a bath containing a hypotonic Ca-free PEG solution in double-distilled water for 2 min. Salomone et al. [182] carried out a study on 60 rats after facial nerve injury. A 500 mM PEG solution in double-distilled water was applied to the case group for modulation of the Ca. As a result, the axonal diameter of the group treated with PEG was greater than the non-treated group; therefore, they concluded that although this method does not provide significant improvement, PEG fusion slowed down demyelination in the PEG-treated group. Several studies have reviewed the roles of drugs and herbal remedies in nerve regeneration by scavenging free radicals. For instance, thymoquinone (TQ), a bioflavonoid, can be harvested from Nigella sativa (NS) and has beneficial anti-inflammatory, antioxidant, antihyperlipidemic, anti-diabetic, anti-allergic, and hepatoprotective impacts. It was reported that TQ has protective consequences for nerve tissue by acting as a free radical scavenger for radicals released after neural trauma. Concerning this, Sereflican et al. [183] assessed the roles of TQ and methylprednisolone after nerve trauma in 24 rabbits. In the first studied group, TQ was administered at 5 mg/kg/day, and in the second group, methylprednisolone was administered at 1 mg/kg/day for two weeks, once a day. Their study revealed that TQ was slightly better than methylprednisolone in functional nerve recovery (Figure 8).

One of the important innervation regions in the craniofacial area is auditory innervation. Few studies have analyzed the inhibitory effects of some special metals, such as lead (Pb) (in the blood), on the auditory nerve and brainstem (cochlear nucleus region). It was found that the administration of both synthetic and natural antioxidants has impressive effects on the prevention of metal-induced biochemical alterations, but the numbers of clinical studies in this field are still limited. For this purpose, Zucki et al. [184] investigated the auditory system and its maturation, particularly the auditory nerve and brainstem, in rats that were exposed to lead acetate and supplemented with ferrous sulfate as an antioxidant agent. In this study, 15 male rats were exposed to 100 mg/L of lead acetate with ferrous sulfate (20 mg/kg), and 15 rats were exposed to 400 mg/L of lead with ferrous sulfate (20 mg/kg) within their drinking water for 6 weeks. The results indicated that the concentration of blood lead could make auditory maturation slow. Furthermore, ferrous sulfate supplementation could modify these deleterious impacts on the auditory nervous system only at a low lead concentration (100 mg/L).

## 5. Conclusions

Oral and maxillofacial diseases are one of the most common disorders in the world which affect health and economic aspects of life. The application of graft materials and cell therapy strategies in the reconstruction of damaged organs can induce infection and increase the level of ROS products in the tissue environment and immune system of the host body, leading to the low-quality healing process of oral and maxillofacial defects. Hence, the application of antioxidant materials in combination with various scaffolds and medical hydrogels is highly recommended by scientists. Antioxidants can fight against several free radicals, including superoxide and hydroxyls, both directly and indirectly by altering the intracellular enzymes’ activities. They are classified into natural and synthetic, exogenous and endogenous, and enzymatic and non-enzymatic groups. As it is clear, oral and maxillofacial tissues consist of a wide range of tissues, such as craniofacial bone, periodontium, dental pulp, dentin, enamel, cartilage, and neuron. Both synthetic and natural antioxidant sources, especially polyphenol structures, are applied for large craniofacial bone defects and periodontal defects. To summarize, several studies have demonstrated that the application of antioxidants in different forms and from various sources, either locally or systematically, can keep oral and maxillofacial tissue safe against free radicals and sometimes enhance cell proliferation, differentiation, and tissue regeneration. In addition, more future studies are required to evaluate and confirm the impacts of antioxidant agents as an alternative treatment for damaged tissues.

## Figures and Tables

**Table 1:** The studies reported on the application of antioxidant materials for oral and maxillofacial tissue regeneration.

	Antioxidant Material	Natural/Synthetic	Form/Dose	In Vitro/In Vivo and Targeted Region	Significant Results	Ref
Craniofacial bone	Polyethyleneglycol citrate-co-N-isopropylacry lamid	Synthetic	Scaffold	Human mesenchymal stem cells (hMSCs)/a mouse model with critical defects	Increased osteogenic differentiation of hMSCs/ robust mineralization and osteogenesis in the animal model (Figure 2B)	[65]
	Propolis	Natural	Systemic application (100 mg/kg)	Premaxillary region in rats	Enhanced bone remodeling and formation of new capillaries	[67]
	Gallic acid	Natural	Powder form and liposome form	Calvarial defects of Wistar rats	Formation of new bone	[68]
	Epigallocatechin gallate	Natural	Gel form	Congenital cleft-jaw model (in vivo)	Increased osteogenic properties and bone regeneration	[69]
	*Nigella sativa* (*N. Sativa*)	Natural	Systemically applied with gelatin sponge graft	Calvarial rat defects	New bone tissue formation	[70]
	Oxidized pullulan	Synthetic	Injectable hydrogel	Murine osteoblast precursor cells (MC3T3-E1)	Reduced inflammatory markers, such as IL-6 and IL-1, enhanced osteogenesis	[71]
	Lactoferrin and substance P	Synthetic	Injectable hydrogel	Critical-sized calvarial defects in mice	Enhanced bone defect healing (Figure 2C)	[66]
	Ganoderma lucidum, a kind of mushroom	Natural	Systemic application	Calvarial rat defects	Osteonectin production	[72]
	Melatonin	Natural	Local application	Dental pulp stem cell (DPSCs)/rat calvarial defects	Promoted proliferation and osteogenic differentiation of DPSCs by regulating COX-2/NF-κB/p38/ERK MARK.	[73]
Periodontal tissue	Phelligridin D	Natural	Local application	Human periodontal ligament cell (HPDLCs) induced by glucose oxidative stress	Enhanced osteogenic and cementogenic properties of HPDLCs	[74]
	2,3,5,4′-tetrahydroxystilbene-2-O-β-D-glucoside	Natural	Local application	Human dental pulp stem cells	Increased osteogenic differentiation of hDPSCs and Improved bone formation	[75]
	Resveratrol and celastrol	Natural	Collagen film	Human periodontal ligament fibroblast cells	Increased proliferation of HPDLF cells	[76]
	Genistein	Natural	Systemically intraperitoneal injected	Mice model /human gingival fibroblasts (hGFs)	Protecting hGFs from ROS products, preventing osteoclast differentiation	[77]
	Virgin coconut oil	Natural	Gel form	Wistar rats	Promoted periodontal tissue healing by effecting the level of inflammatory markers, includingTNF-*α* and TGF-*β*1	[78]
	Taurine	Natural	Collagen membrane	Gingival epithelium	Rapid reepithelization in gingiva	[79]
	Salvadora persica/jellyfish collagen	Natural	Scaffolds	hPDLF cells	Enhanced cell proliferation and regeneration	[80]
	Aqueous *Larrea divaricata* Cav	Natural	Scaffolds	3T3 fibroblasts cells	Increased fibroblast proliferation	[81]
	Coenzyme Q10	Natural	Nanoparticles	In vitro and in vivo in human	Enhanced antioxidant capacity of periodontium, facilitated chronic periodontitis treatment	[82]
	Flower micelles	Natural	Injectable gel	In vitro/in vivo in Sprague–Dawley rats with periodontitis	Inhibiting *P. gingivalis*-induced bone loss	[83]
Wound healing	Thymus essential oil	Natural	Phospholipid vesicles	Keratinocytes cells	Enhanced antioxidant and antibacterial effects on damaged oral tissue	[84]
	Pomegranate ingredient (punicalagin) + Zn	Natural	Solution	Human gingival fibroblast	Induced antioxidant effect, enhanced human fibroblast proliferation, and healing of gingiva injury	[85]
	Aloe vera (AV)	Natural	Gel form	Minor aphthous lesions	Decreased lesion pain, wound size, and also period of wound healing	[86]
	Glutathione and chitosan	Natural	Local application	Intraoral incision of rabbits	Regeneration of oral soft tissue	[87]
	Papaya	Natural	Local application	Labial injuries in mandible anterior parts in mice	Increased healing process in oral ulcers in mice, perfect epithelial layer formation and fibrillation	[88]
	Hydroalcoholic extract of pistachio *vera* seeds(PSE)	Natural	Local application	Rat tongue injuries	Enhanced healing of oral wounds/increased fibroblast proliferation	[89]
	Calendula officinalis (C. officinalis)	Natural	Local application	Injuries in buccal mucosa in Wistar rats	Improved wound healing	[90]
	Curcumin	Natural	Liposomal formulation	Dental pulp stem cells	Increased cell proliferation/inhibition of inflammatory cytokines released through the NFkB/ERK and pERK signaling cascades	[91]
	Ceria nanoparticles	Synthetic	Nanoparticle form	hCPSCs cells	Improved odontoblastic differentiation of hDPSCs, reduced high levels of intracellular ROS in hDPSCs	[92]
	4-Hexylresorcinol	Natural	Ointment form	Dental pulp cells/in vivo test in rat model	Reduced inflammatory cytokines, such as TNF-α and IL-1β, and increased antioxidant capacities and activities	[93]
	Astaxanthin /Fish oil	Natural	Systemic application	In vivo rat model	Keeping pulp tissue safe from oxidative stress-related diseases	[94]
	Amino acid, *N*-acetylcysteine	Natural	Local application (form of powder and liquid)	Rat dental pulp cell extract	Increased cell proliferation and attachment, improved cellular redox system	[95]
	Carrageenan + Cossus quadrangularis, a natural agent	Natural	Injectable hydrogel	hDPCSs cells	Induced dentin regeneration for pulp tissue	[96]
	Chrysin (a kind of plant flavonoid)	Natural	Scaffold	hDPCSs cells	Increased cell viability, reduced level of TNFα, induced anti-oxidant and anti-inflammatory effects, developed mineralization in the dentin-pulp complex	[97]
	Vitamin E alpha-tocopherol (α-T)	Natural	Local application	Odontoblast-like MDPC-23 cells	Decreased ROS products	[98]
